# Training T-shaped translational scientists

**DOI:** 10.1017/cts.2024.674

**Published:** 2024-12-16

**Authors:** Molly Wasko, Kathryn Allen Nearing, Stacey L. Neves, Amy Carrillo, Julie Rainwater, Jennifer A. Croker, Robert P. Kimberly

**Affiliations:** 1 Center for Clinical and Translational Science, University of Alabama at Birmingham, Birmingham, AL, USA; 2 Division of Geriatric Medicine, School of Medicine, University of Colorado Anschutz Medical Campus, Aurora, CO, USA; 3 Rocky Mountain Regional VA Medical Center, VA Eastern Colorado Geriatric Research Education and Clinical Center (GRECC), Aurora, CO, USA; 4 School of Medicine, Office of Research, University of California Davis, Sacramento, CA, USA

**Keywords:** Translational scientist, workforce development, I-Corps, T-shaped scientist, customer discovery

## Abstract

To evaluate the design of I-Corps@NCATS as a translational scientist training program, we mapped specific elements of the program’s content and pedagogy to the characteristics of a translational scientist, as first defined by Gilliland et al. []: systems thinker, process innovator, boundary spanner, team player, and skilled communicator. Using a mixed-methods evaluation, we examined how the I-Corps@NCATS training program, delivered across twenty-two Clinical and Translational Science Award Hubs, impacted the development of these key translational scientist characteristics.

**Methods:**

We developed survey items to assess the characteristics of systems thinker, process innovator, boundary spanner, team player, and skilled communicator. Data were collected from a national sample of 281 participants in the I-Corps@NCATS program. Using post-then-retrospective-pre survey items, participants self-reported their ability to perform skills associated with each of the translational scientist characteristics. Additionally, two open-ended survey questions explored how the program shifted participants’ translational orientation, generating 211 comments. These comments were coded through a team-based, iterative process.

**Results:**

Respondents reported the greatest increases in self-assessed abilities related to systems thinking and skilled communication. Participants indicated the highest levels of abilities related to team player and boundary crosser. From the coding of open-ended comments, we identified two additional characteristics of translational scientists: intellectual humility and cognitive flexibility.

**Conclusions:**

Participation in I-Corps@NCATS accelerates translational science in two ways: 1) by teaching the process of scientific translation from research ideas to real-world solutions, and 2) by encouraging growth in the mindset and characteristics of a translational scientist.

## Introduction

In the context of biomedical research, translation represents the process of advancing ideas across boundaries from scientific discovery – whether from the laboratory, the clinic, or the community – to changes in clinical practice through the development of new drugs, diagnostics, devices, novel interventions or lifestyle modifications [1]. Core to translational science is the concept that scientific discoveries must be transferred, or “translated,” across different phases of research, moving from basic science to human studies. This process is often represented by a continuum from laboratory-based mechanistic studies (T0) through various stages of human application: T1 (translation to humans), T2 (translation to patients), T3 (translation to clinical practice), and T4 (translation to the community). These stages are designed to inform clinical guidelines and ultimately improve human health [2]. A skilled and knowledgeable scientific workforce is essential for successful research translation. These scientists not only advance knowledge within their specific fields but also facilitate the transfer of discoveries across research phases, from the laboratory to clinical practice [3]. A key challenge in translational science is how to intentionally design training programs that adapt traditional curricular approaches in new ways to develop the unique perspectives and skills required of a translational scientist.

In 2017, the National Center for Advancing Translational Sciences (NCATS) funded the development of an introductory research commercialization short course, called I-Corps@NCATS, modeled after the National Science Foundation’s successful I-Corps program. Building upon a two-year I-Corps@NCATS pilot study involving nine Clinical and Translational Science Award (CTSA) Hubs, I-Corps@NCATS was disseminated to an additional 13 partner Hubs from 2020-2023 (see Appendix A for the list of participating CTSA Hubs). The primary goal of I-Corps@NCATS is to train clinical and translational scientists in the process of customer discovery to assess the clinical need and market potential of research innovations. A longer-term goal of I-Corps@NCATS (beyond the scope of this study) is to accelerate the translation of scientific discoveries from lab to market to improve patient care through research dissemination, implementation, and commercialization.

**Figure 1.**
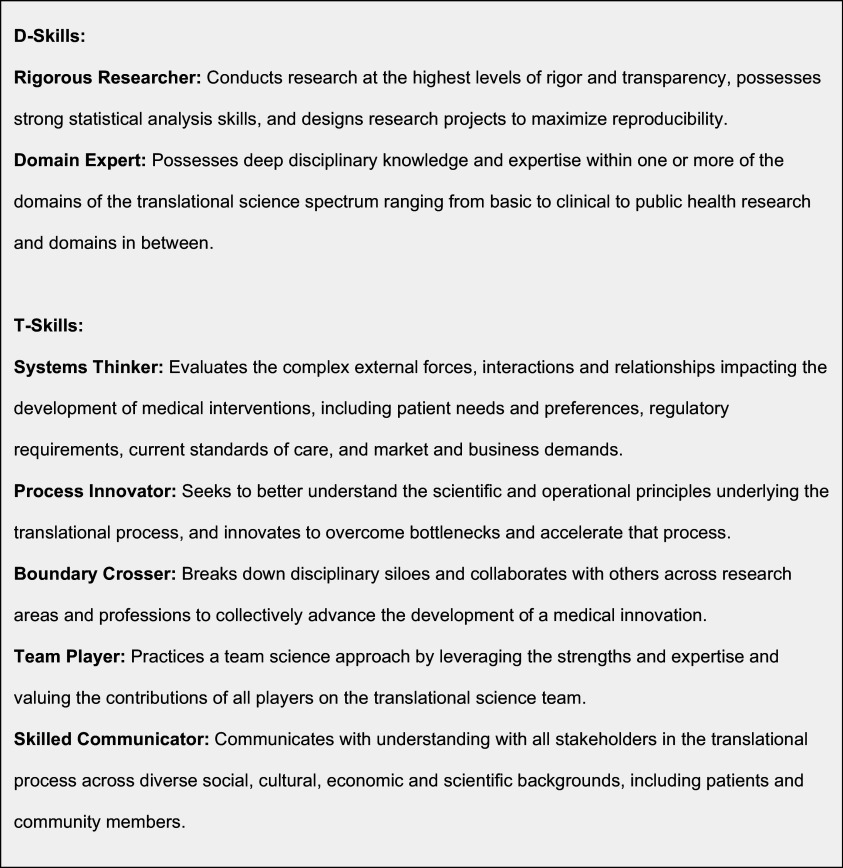
Distinguishing characteristics of a translational scientist (from Gilliland et al. 2019).

**Figure 2.**
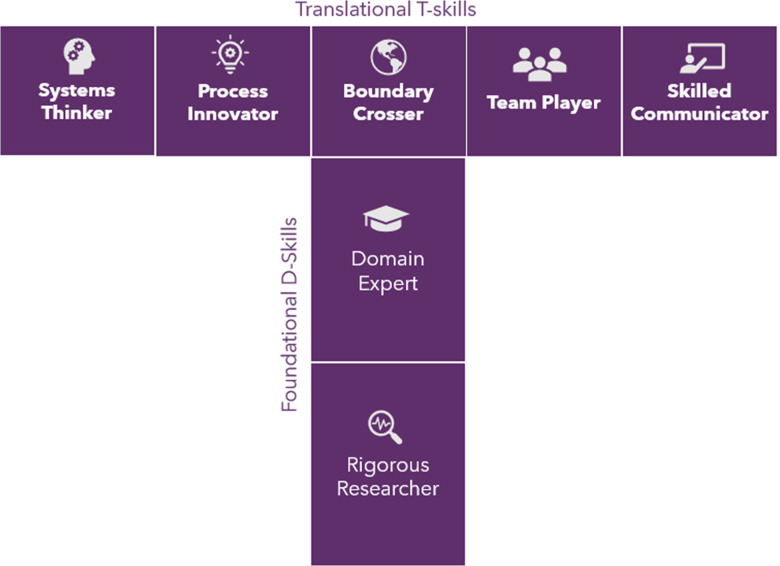
The T-shaped scientist: Foundational D-skills with translational T-skill.

A key observation from the pilot program was that many of the participants exhibited a significant change in mindset towards their approach to research [4]. This observation generated the hypothesis that participation in the I-Corps@NCATS program is an effective way to develop the characteristics of translational scientists described by Gilliland *et al*. [5] A translational scientist is a rigorous researcher with deep domain expertise, who also possesses qualities such as systems thinking, process innovation, boundary crossing, teamwork, and strong communication skills (see Fig. 1). Building on this framework, we distinguish between “D-skills,” which represent deep, discipline-based or disease-specific expertise, and “T-skills,” which involve the ability to transfer knowledge and solutions across the translational spectrum (e.g., translation, transformation, teams) [6]. The concept of a T-shaped scientist depicts a researcher who not only excels in a specific discipline (the vertical part of the “T”) but also integrates knowledge across various domains and stages of translation (the horizontal part of the “T”). While similar to conventional scientists in their strong disciplinary foundation, T-shaped scientists go further by demonstrating the ability to bridge disciplines and integrate knowledge across domains and translational phases (see Fig. 2). To evaluate the impact of the I-Corps@NCATS program, we mapped its curriculum components to the characteristics of a translational scientist and measured changes in participants’ self-perceived abilities before and after the program.

### I-Corps@NCATS course design and translational scientist characteristics

The primary educational elements of the I-Corps@NCATS program are the integration of content (the subject matter) and pedagogy (the methods of teaching and learning).

#### Content

The specific content domain of I-Corps@NCATS is entrepreneurship with emphasis on the commercialization of scientific discoveries. The program covers three main topics: 1) startup fundamentals, 2) the Business Model Canvas [7], and 3) customer discovery. Participants apply this learning to validate problem-solution fit: the identification of a specific customer segment with an unmet need that the innovation is uniquely positioned to address (value proposition). By the end of the program, participants should be able to use the Business Model Canvas as a tool to develop and test hypotheses about customer segments and value propositions, create an ecosystem map to identify various customer roles within a customer segment (e.g. patient, provider and payer), and conduct customer discovery through direct interviews with potential customers and stakeholders.

#### Pedagogy

Key elements of the I-Corps@NCATS pedagogy, designed to enhance engagement, motivation, and knowledge retention, include:

1. Team Participation: Participants enroll as teams, which allows members to share the workload and engage in collaborative problem-solving. These teams work on their own research innovations, enhancing the relevance of the course material by making the content directly applicable to their specific research objectives.
2. Experiential Learning: Teams engage in “learning by doing” through customer discovery, guided by the instructors. The immersive five-week schedule balances content delivery, interviews, presentations, and coaching to reflect the time pressures and intensity of entrepreneurship.
3. Inquiry-Based Learning: Teams test assumptions about customers and innovations through direct interviews, focusing on customer needs. Participants conduct at least 30 interviews to gather data, and then work together as a team to interpret findings and refine their approach.
4. Socratic Method: Instructors engage the teams in dialog during presentations, encouraging critical thinking by questioning insights and emphasizing data-driven conclusions. This fosters reflection and shared learning across all teams.

**Figure 3.**
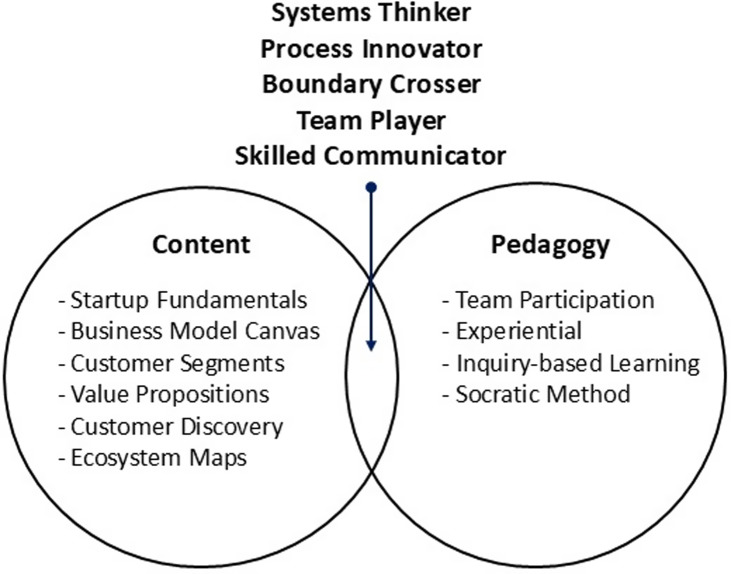
Summary of I-Corps@NCATS content and pedagogy.

We anticipated that the combination of I-Corps@NCATS content and pedagogy would enhance participants’ development of T-skills. For example, teams map workflows and ecosystems to identify how and for whom their solution could improve or disrupt current processes, building systems-thinking and process innovation skills. Completing 30 customer discovery interviews in five weeks pushes teams to leave their labs and engage with end-users, decision-makers, influencers, and potential competitors outside their usual networks. This shift from expert to novice to seek information from diverse stakeholders fosters curiosity and encourages participants to ask open-ended questions, helping them develop boundary-crossing skills. Organizing and managing the workload, interpreting interview findings, and making sense of interview data in the context of the team’s research discovery builds teamwork skills. The interviews and weekly presentations provide opportunities to practice communicating about science in the language of stakeholders and the language of I-Corps and entrepreneurship, building communication skills (see Fig. 3). Detailed connections between the I-Corps@NCATS content (Appendix B), pedagogy (Appendix C), and the characteristics of translational scientists are provided in the Supplementary Materials.

### Data collection and methods

The I-Corps@NCATS program and its evaluation have been described in detail in prior publications [4,8–11]. The data for this study were collected through a national mixed-methods evaluation, which included a post-program survey administered to all participants at the end of the final training session. To improve response rates, at least two reminders were sent to non-respondents over two weeks. QualtricsXM software was used to develop and administer the surveys. A total of 30 cohorts, comprised of 214 teams and 568 unique participants completed the I-Corps@NCATS short course from May 2020 through May 2023. Table 1 shows the distribution of participants across programs. The study sample consists of individuals who completed I-Corps@NCATS and responded to the post-training survey items (*n* = 281). On average, we received survey responses from 54% of the participants and 89% of the teams.

**Table 1.** I-Corps@NCATS participating sites, program, teams, and participants

Host Site	# of Programs	Teams	Participants
National Program (All Partner Hubs)	1	4	16
Case Western Reserve University	3	32	82
Columbia University	3	11	27
Loyola University Chicago	3	17	55
Medical College of Wisconsin	1	9	25
Northwestern University	2	13	41
Oregon Health & Science University	2	17	46
Pennsylvania State University	4	18	37
University of Rochester	1	7	13
University of Buffalo	1	7	22
University of California, Davis	2	12	19
University of Colorado, Denver	4	17	61
University of Miami	3	29	75
University of Virginia	2	9	28
University of Massachusetts	2	12	21
TOTAL Completers**		**214**	568**

** Includes 6 individuals that participated in more than one program.

**Figure 4.**
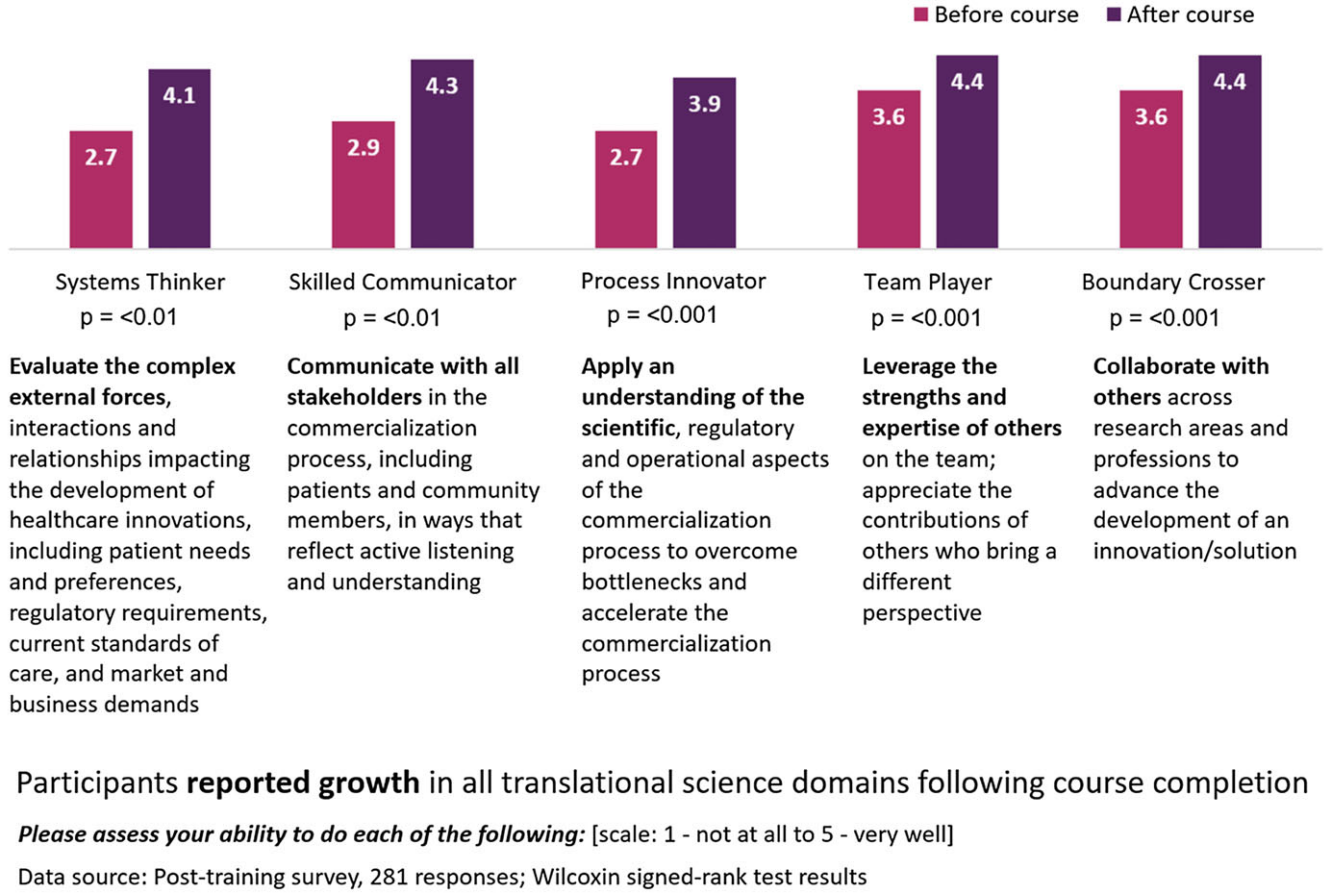
T-skills: Post-then-retrospective-pre self-assessment survey results.

Items assessing the T-skills were included in the post-program survey. Participant’s self-reported ability to perform each of the translational skills was measured with a post-then-retrospective-pre survey item [12,13] using a Likert scale of 1–5, with 1 indicating not at all and 5 indicating very well. A post-then-retrospective-pre survey item was chosen to increase validity of the self-reported data by minimizing response-shift bias. This bias can occur when participants gain a better understanding of the concepts or become aware of knowledge gaps that they did not recognize prior to the training. In wording the survey items, we sought to achieve a balance between maintaining fidelity to the original language of the characteristics, alignment with I-Corps@NCATS terminology, and balancing brevity with clarity for survey administration purposes. Eight site leads involved in the I-Corps@NCATS pilot reviewed and provided feedback on the modified language. The wording of the final items is provided in Fig. 4. In addition to the T-skills survey items, two open-ended questions (i.e., : “*Please share how the customer discovery process shaped your experience during the program.*” and “*How has participation in the I-Corps@NCATS program affected your entrepreneurial mindset and networks?*”) provided additional opportunity to validate that participants were describing changes in translational skills as a result of the program experience and the impact of the program on professional development.

### Data analysis

To determine whether there was a significant difference in post-then-retrospective-pre self-assessment ratings, we conducted a Wilcoxon signed-rank test using IBM SPSS software, version 28 (IBM Corp., Armonk, NY). We selected the Wilcoxon signed-rank test—the non-parametric equivalent of a paired samples *t*-test—as the data showed substantial departure from normality [14]. In addition to quantitative analysis, we conducted thematic analysis of the responses to the two open-ended survey items. To support a systematic, team-based approach to analyzing the open-ended responses, we developed a codebook featuring codes and an operational definition of each through iterative cycles of reviewing the data and team processing discussions. Codes were derived both deductively, based on published characteristics of a translational scientist, and inductively through new insights facilitated by bi-monthly meetings. For codes that emerged inductively, operational definitions were determined through discussions among coders.

## Results

### Development of translational T-skills – quantitative survey results

The results indicated a significant increase in all five characteristics (significance level (*α*) was set at 0.05.), and the results are presented in Fig. 4. Effect sizes ranged from moderate to large, signifying practical significance. The greatest increases in self-reported abilities were associated with systems thinking (from 2.7 to 4.1) and skilled communication (from 2.9 to 4.3). Participants indicated the highest levels of ability in relation to being a team player and boundary crosser (both rated 4.4 out of 5).

### Development of translational T-skills – qualitative results

Responses to the open-ended question, *“Please share how the customer discovery process shaped your experience during the program,”* revealed ways that I-Corps@NCATS shaped the development of translational T-skills. Respondents described gaining a greater appreciation for and knowledge of the complex array of factors influencing efforts to bring a given solution to market, which required systems thinking.

One respondent explained:

“… We uncovered the unforeseen existence of potential bottlenecks such as hospital administration and medical insurance that need to agree and [the need to] establish a contract before a costly therapy can be reimbursed. The customer discovery process was an eye-opener for us and definitely made us rethink how to optimize our product so it can be adopted more easily …”

Another respondent stated:

“[I-Corps@NCATS] forced us to think through the ecosystem of payers, customers, partners, collaborators, referral providers, etc.”

Other participants specifically commented on ways that identifying individuals to interview as part of the customer discovery process tested their ability to span boundaries (i.e., to get out of academic siloes and network across sectors). This led to invaluable insights regarding problem-solution-fit and supported process innovation.

One respondent explained:

“Customer discovery allowed us to understand how our [solution] fit into other customer segments’ day to day operations. It allowed us to hone in on a product that will meet others’ definitions of success and meet their needs.”

For another respondent, spanning discipline-specific boundaries allowed them to explore more broadly for whom a given solution would benefit and under what conditions:

“[Spanning boundaries] caused me to realize obstetricians would be an excellent beach head [market]. [It] taught me [the] value of direct cost savings versus indirect benefits of reducing complications.”

Still, another respondent stated:

“[Customer discovery] gave us more information about what motivates [customers in] different industries, which helps define boundaries for the problem-solution fit.”

### Additional translational T-skills – intellectual humility and cognitive flexibility

Our data analysis identified two additional translational scientist characteristics: intellectual humility and cognitive flexibility. These insights emerged from our direct observations of participants and subsequent data analysis. Intellectual humility is an interpersonal characteristic that involves the recognition that one’s beliefs are fallible. Intellectual humility has been defined as “the degree to which people acknowledge that [what] they believe to be true may, in fact, be incorrect” [15]. Intellectual humility involves an appreciation of others’ strengths and contributions, and realization that the increased “unpredictability and unknowability” in modern science requires researchers with “more humility and less hubris” [16]. The following response is illustrative of how intellectual humility allowed participants to guard against confirmation bias, fostered intellectual curiosity, and created openness/receptiveness to new information and insights:

“We ha[d] a methodology based on the belie[f] you needed to engage the customer by bringing information and features about the product. With the interviews, we [found] the opposite is more beneficial: you only need to ask questions …, and then let the customer give you information and you give yourself time to listen.”

Cognitive flexibility is described as “the ability to switch cognitive sets to adapt to changing environmental stimuli” [17] and allows individuals to shift their thinking and change direction based on new information [18]. Cognitive flexibility also enables individuals to produce creative solutions by combining knowledge or skills from diverse or seemingly unrelated domains, by taking the perspective of another to see the value of an innovation from their point of view. In the context of translational thinking, we define cognitive flexibility as an individual’s ability to recognize, interpret, and integrate new information, alter existing perspectives, and engage in new behaviors. Examples of cognitive flexibility included comments like,

“It exposed the misconceptions we had. It helped us pivot to an entirely different beach head market with confidence.” Cognitive flexibility was reflected in participants’ willingness to work to “identify the needs of customers and adapt our business model according to those needs.”

Underscoring the importance of I-Corps@NCATS as a training program targeting the development of a specialized translational science workforce, comments also frequently documented how the program contributed to changes in mindset and impacts on professional development in general, not just in the context of entrepreneurship or commercialization. Such comments included,

“I am starting to think differently;” and

“I am thinking [of] applying concepts to current programs and other implementations in my research.”

One respondent stated,

“Honestly, I [had] no previous experience in entrepreneurship. The program help[ed] me develop an insight in entrepreneurship and force[d] me to step out of my comfort zone to build my network. This is really helpful for my career development.” Another respondent shared, “I learned skills … and ways to avoid the typical pitfalls that cause startups to fail. I’m hopeful for the future of this project but, regardless, I will use these skills throughout my career.”

See Appendix D for illustrative feedback from the qualitative coding.

## Discussion

I-Corps@NCATS is a training program designed specifically for academic investigators to introduce them to the basics of entrepreneurship and how to assess the market potential of their research discoveries. Findings from the national mixed-methods evaluation indicate that the I-Corps@NCATS program significantly enhanced the development of the five key characteristics of a translational (T-shaped) scientist. This highlights a crucial takeaway: translational T-skills can be learned, and training programs can be intentionally designed to develop T-shaped scientists. This study emanated from observations from the field to test a “hunch” about how and why the I-Corps@NCATS program seemed to have such a significant impact on participants’ thinking. This is an example of how research questions gained from observations in a later translation phase, such as T3 translation to practice, can inform earlier translational phases and contribute to basic, fundamental research questions. The field presents a dynamic environment where unexpected phenomena can be observed. These unexpected observations often lead to new hypotheses and research questions that might not arise through theoretical or laboratory work alone. Similarly, field observations through customer discovery allow participants to experience the environment where their research solutions will be applied, uncovering unexpected phenomena, limitations, or emerging trends.

By participating in this program, scientists recognize that they are better prepared to broaden their focus beyond the lab and make a real-world impact with their research. The evaluation survey items related to the translational scientist characteristics, that assess actual changes in participants’ skill development, enable participants to better recognize and appreciate their personal and professional growth, which can often go unnoticed without a structured approach to self-reflection. Additional findings from the qualitative analysis suggest two additional characteristics – intellectual humility and cognitive flexibility. Intellectual humility, or being open to different ideas, is essential for respectful engagement with patients and communities, while cognitive flexibility, the ability to integrate new ideas into one’s thinking and practice, enhances empathy and perspective-taking. These skills are critical not only for entrepreneurs but also for creating an innovative research workforce that is empathetic and responsive to societal needs. Together, these two traits, alongside the original five T-skills, can provide scientists with the curiosity, motivation, and skills needed to bridge boundaries and engage with diverse perspectives, enriching their research and making it more inclusive.

Results indicate that the program facilitates development of the attitudes, knowledge, and skills needed to navigate the increasingly complex academic biomedical research ecosystem. Translational scientists excel at considering and communicating their science from multiple viewpoints, a skill that can be built into many workforce development programs by encouraging scientists to engage directly with the people their innovations aim to help. Success in this environment depends not just on pitching ideas but on being open to pivoting based on new information and changing contexts. Some participants reported intentions to apply customer discovery as a repeatable method to other translational research endeavors as a way to generate new insights that can inform future research directions. The development of these skills could be designed into many of our translational workforce development programs by encouraging scientists to “get out of the lab” and learn directly from the people that an innovation is intended to benefit. This leads to the final contribution from this study: “T-skills” are valuable not only for commercially oriented scientists but also for academically focused investigators. Strategic, long-term investment in programs like I-Corps@NCATS, that pull our investigators out of the lab, is necessary to achieve a more innovative research workforce that is empathetic and responsive to the needs of society.

### Limitations

This study should be viewed as an initial attempt to test the hypothesis that programs like I-Corps@NCATS can effectively and intentionally promote the development of T-shaped translational scientist characteristics. While the results are promising, several limitations need to be considered. First, the study used a single survey item to quantitatively assess self-reported gains in participants’ abilities related to the characteristics of a translational scientist. Future research should focus on developing multi-item, validated scales to ensure reliability and establish convergent and discriminant validity. Such scales are critical for evaluating the effectiveness of translational workforce development programs, for comparing outcomes across different programs, and for offering participants a valuable tool for self-reflection. Second, future research should explore the construct validity of the newly identified characteristics, intellectual humility, and cognitive flexibility, and develop a theoretical framework to incorporate these characteristics. For example, it remains to be determined whether these are independent constructs to be added to the horizontal part of the “T” of a T-shaped scientist, or if they should be added to the list of “D” skills as characteristics of rigorous researchers. Additionally, more research is needed to examine the interplay among the characteristics to identify whether there are interdependencies, such that some characteristics act as intermediate skills that reinforce the development of others. Lastly, the generalizability of the pedagogical approaches used in I-Corps@NCATS to other training programs is uncertain. While elements like team participation, experiential, and inquiry-based learning may be relevant to translational scientist training programs, this assumption needs to be tested through future research.

## Conclusion

This research builds on the concept of a T-shaped translational scientist, who combines deep knowledge and disciplinary expertise (D-skills, represented by the vertical part of the “T”) with skills like skilled communications, teamwork, boundary spanning, process innovation, and systems thinking (T-skills, represented by the horizontal part of the “T”). Additionally, we propose that T-shaped scientists also exhibit the characteristics of intellectual humility and cognitive flexibility. This work has two key contributions. First, it demonstrates that training programs can be designed to develop translational skills, providing lessons that can be applied to other translational science workforce development programs. Second, it highlights the value of using a scale to assess the effectiveness of these programs in fostering translational science skills. By improving our understanding of how to design programs that address both traditional and translational skills, our goal is to accelerate the development of a specialized translational science workforce that can flexibly adapt to novel situations and generate innovative ideas within and across domains, ultimately advancing the goals of translational science.

## Supporting information

Wasko et al. supplementary material Wasko et al. supplementary material

## Supplementary material

The supplementary material for this article can be found at https://doi.org/10.1017/cts.2024.674.
