# Supplementary material for: Training T-shaped translational scientists
Source: J Clin Transl Sci. 2024 Dec 16;9(1):e5. doi: 10.1017/cts.2024.674 (PMC11736293; doi:10.1017/cts.2024.674)
Supplement: Wasko et al. supplementary material [file S2059866124006745sup001.docx]

**Appendix A: I-Corps@NCATS National network of partnering institutions**


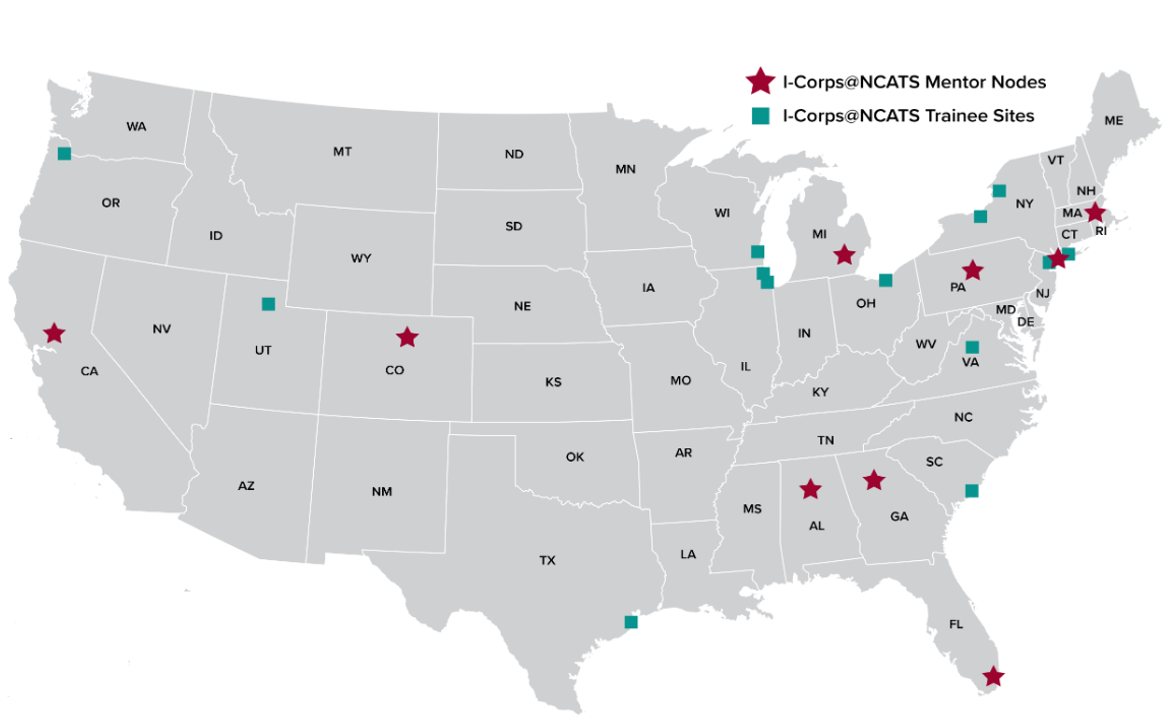


| **Mentor Nodes** | **Trainee Sites** |
| --- | --- |
| Emory University/Georgia Tech | Case Western Reserve University |
| Pennsylvania State University | Columbia University |
| Rockefeller University | Loyola University Chicago |
| University of Alabama at Birmingham | Medical College of Wisconsin |
| University of California, Davis | Medical University of South Carolina |
| University of Colorado, Denver | Northwestern University at Chicago |
| University of Massachusetts | Oregon Health & Science University |
| University of Miami | Rutgers University |
| University of Michigan | University of Texas Medical Branch, Galveston |
|  | University of Buffalo |
|  | University of Rochester |
|  | University of Utah |
|  | University of Virginia |

**Appendix B: I-Corps@NCATS training content aligned to translational scientist characteristics**

| **Core Component – Customer Discovery Methodology**  **What we teach** | **Objectives (Goals for Learners)** | **Translational Scientist Character Traits** |
| --- | --- | --- |
| 1. Customer discovery is a rigorous, structured, repeatable process that follows the scientific method | - Evidence driven – *Did you hear that from a customer or is that something you believe?* - Rigorous process focused on systematically disproving hypotheses as a guard against confirmation bias | Vertical skills (D-shaped scientist)  Scientific humility* |
| 1. Customer discovery involves the systematic exploration of evidence that challenges your biases and assumptions; hypotheses or business theses are revised iteratively in response | - Rigorous process that involves exploring contradictory evidence - Generating and testing hypotheses by talking with customers - Summarize findings in a figure (ecosystem map) - Secondary research (not reviewing the literature on the relevant science, but to understand a given industry and the ecosystem in which an innovation may be introduced) – different facet of D-shaped scientists’ deep understanding | Vertical skills (D-shaped scientist)  Systems thinker  Skilled communicator (can communicate information in different ways for different audiences, including visually through diagrams) |
| 1. This is a process of discovery, not pitching or selling the solution. | - Focus of short course: discovering who their customers are, their customers’ needs and how the customer is addressing those needs (or not) now. | Scientific humility*  Skilled communicator (ability to listen, seeking understanding)  Cognitive flexibility (flexible thinking and ability to pivot based on evidence)* |
| 1. Talk to people you don’t know. Get out of the building and off campus (avoids confirmation bias) | - Learn how to talk to strangers to network, listen and learn from customers and other stakeholders for discovery - Understanding that there is a broader landscape of who you should be talking to (who are the different stakeholders, what are their roles, what are their jobs; explore how customers outside of their organization solve this problem now and do they think it is a pain point - How to create an ecosystem or process map as a tool for identifying stakeholders (customer roles) and how a solution may disrupt current systems/processes | Skilled communicator  Boundary Spanner  Systems thinker  Process Innovator |
| 1. Ask open-ended questions -- cannot be answered with a yes/no; probe for more description to keep exploring the why   It’s ok to say, “I don’t know” in this course. | - Hear what the interviewee is thinking, explore customer experience (as opposed to the interviewer driving the conversation by asking leading questions) - Value the customer experience and perspective as much as their own scientific knowledge - Reinforce that we learn more by listening than talking | Skilled communicator  Boundary Crosser  Scientific humility* |
| 1. Iterative -- what is learned from initial interviews informs new questions explored in subsequent interviews; determine who to interview based on new information/questions   Ability to pivot or make a “no-go” decision based on emerging evidence | - Identify patterns in data and what this indicates about the ecosystem in which the customer/key decision maker is operating and how this context might influence adoption decisions - An n=1 is not enough data to test a hypothesis - Allowing your data to lead you rather than your preconceived notions to drive your research | Systems thinker  Cognitive flexibility* |
| 1. Interviews explicitly inform completion of business model canvas. Customer segments and Value propositions should become increasingly specific. | - Ability to identify patterns in data - Transparency and reproducibility | Cognitive flexibility*  Boundary crosser (become a domain expert in the customers’ domain)  Systems thinker |
| 1. "How-tos” of interviewing. Examples include: generating leads (e.g., using LinkedIn, members of Teaching Team, etc.), value of having two members of team participate in each interview, doing more listening than talking, good question/bad question exercise, etc. | - How to build your network - How to interview - How to use interview data to disconfirm/refine hypotheses and generate new hypotheses - Must work as a team to get the interview (each person leveraging their networks), conduct the interview (one person interviewing, the other taking notes and helping to probe), analyze interview data to determine themes | Team Player  Skilled Communicator |

**Appendix C: Aspects of I-Corps@NCATS pedagogical approach and how each teaching method supports the development of a translational scientist**

| **Core/Essential Component** | **Description/Example** | **Rationale/Purpose (how supports)** | **Translational Scientist Characteristic** |
| --- | --- | --- | --- |
| Uses coaching versus consulting | Members of teaching team ask generative questions. Resist the urge to provide answers/fix/rescue.    Coach how to think, not what to think. | Encourages participants to rely on the customer discovery process as the source of learning, with answers (insights) emerging from customer interviews. Reinforces essential importance of engaging the customer/key decision maker. | **Systems Thinker:**   - Learning from customers to gain deeper understanding of translational contexts and processes and where solution can add value.   **Process Innovator:**   - Discovering the complex array of factors influencing uptake of a new innovation |
| Teaching to the room to keep all teams engaged | Teams are comprised of at least 2 individuals. Member in earlier stage of career encouraged to take the lead.  Multiple members of teams participate in interviews (data collection and analysis), present and respond to questions. | Entrepreneurship is a type of translational science that requires well-functioning multi-disciplinary/trans-disciplinary teams.  Creates a learning environment in which teams are learning from one another. Maintains intensity of engagement throughout. Optimizes learning and opportunities to reinforce key messages. | **Boundary Crosser and Team Player**   - Ability to work with others across research areas and sectors. Requires taking different point of view (e.g., of customer archetype) to gain novel insights re: problem-solution-fit.   **Systems thinker**   - Diverse perspectives support consideration of broader array of factors, implications |
| Interrupting with a purpose to  create teachable moments | Interrupting used to make a key point, reinforce a key learning/ insight, teach to the room. May also be used to keep focus on customer discovery and to avoid “tech talk.”  Teaching team asks questions during presentations to surface/reinforce key points about the process that are relevant to all teams, not just the presenting team. | Allows teaching team to reinforce core concepts, key messages.  Acclimates participants to culture of entrepreneurship (rapid pace, direct, focused; prep for the pitch) | **Skilled communicator:** modeling skilled communication (clear, succinct, actionable; emphasis: avoiding jargon and acronyms) |
| Deemphasizes importance of solution (at customer discovery phase) | Invites just enough information about innovation to provide context to ensure participants recognize, respond to insights from customer discovery.  Teaching team saves questions/discussions about tech for office hours/ coaching sessions. | Maintains the focus: who is the customer, what is the value to that customer. Design with the end-user in mind (customer focused rather than feature focused).  Reinforces: you don’t know what you don’t know. | **Systems Thinker:**  Learning from customers to gain deeper understanding of translational contexts and processes. Discovering opportunities and constraints of customer based on larger context |
| Team report-outs rather than presentations | Report-outs are structured, with reporting templates provided; short, timed and interrupted.    Teaching team is looking for evidence of fidelity to customer discovery process and listening for teachable moments – opportunities to reinforce key messages of the I-Corps@NCATS training program. | Maintains the focus on customer discovery – progress in conducting interviews, resulting insights, next steps. Teaching team gives pointed feedback. | **Skilled Communicator:** Ability to communicate effectively (clear, succinct, minimizing use of jargon)  **Systems Thinker:** Evaluate the complex external forces, interactions and relationships impacting the development of healthcare innovations, including patient needs and preferences, regulatory requirements, current standards of care, and market and business demands  **Process Innovator:** Apply an understanding of the scientific, regulatory and operational aspects of the commercialization process (i.e., innovate) to identify and overcome bottlenecks and accelerate the commercialization process |
| Intensively and intentionally immersive | Teams are expected to conduct and analyze 30 interviews within the 5-week short course, with interviews leading to the iterative development and refinement of hypotheses re: value propositions and business thesis. | Requires immersion throughout iterative rounds of data collection and analysis to maximize opportunities for learning  Requires maintaining the intensive drive and focus of an entrepreneur. | **Rigorous researcher:** conducts research at higher levels of rigor and transparency, possesses strong analytical skills |
| Relies on scientific method | Teaching team anchors work in scientific method: theory is built through generating and systematically working to disprove hypotheses.  Teaching Team reorients teams from proving the value to discovering the value of their solution  Participants are coached to take naïve stance and approach customer discovery with openness to new learning that challenges previously held ideas/conceptions about innovation | Uses a rigorous, repeatable process that is familiar and teams can apply to any project  Helps guard against confirmation bias | **Rigorous researcher:** conducts research at higher levels of rigor and transparency |

**Appendix D: Codebook and frequency distribution of applying codes during analysis of open-ended responses**

| **Code (# of open-ended survey responses coded)** | **Operational Definition** | **Illustrative Quote** |
| --- | --- | --- |
| Characteristics of a T-shaped Translational Scientist | | |
| Intellectual Humility  (n= 87) | The degree to which people acknowledge that that which they believe to be true may, in fact, be incorrect. | “I am a scientist without any experience outside the lab in translating research into a useful product. This program helped me dive into how customer discovery works and understand the huge gap between our expectation of the market and what customers actually want.” |
| Cognitive Flexibility  (n=27) | The ability to recognize, interpret and integrate new information, alter existing perspectives and engage in new behaviors | - “I found out that my real customers are not the ones I thought at the beginning, which completely changed the entire project hypothesis. It was extremely useful.” - “The customer discovery process led us down a variety of different avenues as far as applications, leaving us with a vastly changed hypothesis and a lot of potential approaches to take, which helps to de-risk our venture.” |
| Systems Thinker  (n=37) | Evaluates the complex external forces, interactions and relationships impacting the development of medical interventions, including patient needs and preferences, regulatory requirements, current standards of care, and market and business demands | “… We became aware of the marketing aspect of proceeding with a biomedical product. Who makes the decision to buy it, and how does it compare to the existing technology, the competition? Does the product make the customer’ preparation/ work better? Are insurance companies involved? Can the product be produced at a reasonable or competitive price?” |
| Process Innovator  (n=8) | Apply an understanding of the scientific, regulatory and operational aspects of the commercialization process to overcome bottlenecks and accelerate the commercialization process | “I had a very broad perspective of my technology at the beginning of the program. I foresaw its use in academia, hospitals, and a variety of other institutions. Through interviewing potential customers, I realized that there is a precise location that would be best suited for my technology. I gained a thorough understanding of the field I am trying to develop tech for.” |
| Boundary Crosser  (n=30) | Breaks down disciplinary siloes and collaborates with others across research areas and professions to collectively advance the development of a medical innovation | - “Starting the program, we had no idea what to do, where to begin or what to expect. Going through this process was a labor of love and allowed a greater depth of understanding for our team around the CBRN and the needs from both the researcher and [community-based organization] side.” - “Academic mindset is different from going into the target population and asking them the actual problem.” |
| Team Player  (n=5) | Practices a team science approach by leveraging the strengths and expertise and valuing the contributions of all players on the translational science team | - “The connections with the Teaching Team and customers open[ed] topics that I would have never thought of.” - “The targeted, focused nature of this particular program was timely and very educational to me. We had 3 instructors with different experiences and insights; yet, all provided a structure to the customer discovery process.” |
| Skilled Communicator  (n=25) | Communicates with understanding with all stakeholders in the translational process across diverse social, cultural, economic and scientific backgrounds, including patients and community members | - “Speaking with people who will use the technology gave us the language to speak more specifically about our topic and to ask better questions.” - “… I learned how to ask open-ended questions … I learned how to extract substantially more information during interviews, get stories knowing that means I made a real connection and the person was comfortable, and referrals on who else I can [interview].” |
| Curriculum Design Elements of the I-Corps@NCATS Program | | |
| Hypothesis (value prop) testing  (n=103) | Generating, eliminating and refining value propositions, which evolve over time in direct response to insights from customer discovery interviews. | “Receiving feedback from potential customers strengthened our value proposition and validated the basic concept of our company that point of care testing would be useful.” |
| Conducting interviews  (n= 91) | Pertains to some aspect of scheduling and conducting customer discovery interviews | “Snowball [sampling] was very effective in getting connected to some very good sources (3rd level snowball, especially). Showed [the] power [of] talking with people to understand the problem from many angles.” |
| Problem-solution fit  (n=53) | Understanding the problem from the customer’s perspective, how a solution addresses a customer’s top of the mind needs; how much better the solution needs to be to supplant what customer is already doing/using | “Helped us frame the question our device attempts to solve in a different way. Not just a device looking for a market, but a problem that needs a solution that we can address with technology.” |
| Customer Segment(s)  (n=49) | Referring to identifying a well-defined group of people that have the same needs/job/problem; a logical group that can be served with the same business model. | - “As one of the target customers, I thought that I knew all about the customers. Then I realized that there are many varieties of customers who are out there. We learned various customer segments and pain points that we did not consider initially.” - “Helped understand the customer needs/validated some assumptions and negated others. Identified a new customer we had not considered and ruled out another (found out what would make them interested, though) …” |
| Networking  (n=15) | Intentionally expanding social ties (e.g., to identify individuals to interview) | “It helped me to get out of the lab, extend my comfort zone, and meet with actual end-users who would be using our technology. I had a chance to gain insight from their perspective, as well as what they value and their challenges in the field and regarding new technologies.” |

*Responses could pertain to more than one code. Therefore, the total number of times codes were used (n= 530) exceeds the total number of responses to the corresponding survey item (n= 211).
